# Enhancing generalization in zero-shot multi-label endoscopic instrument classification

**DOI:** 10.1007/s11548-025-03439-5

**Published:** 2025-06-11

**Authors:** Raphaela Maerkl, Tobias Rueckert, David Rauber, Max Gutbrod, Danilo Weber Nunes, Christoph Palm

**Affiliations:** 1 Regensburg Medical Image Computing (ReMIC), OTH Regensburg, 93053 Regensburg, Germany; 2 AKTORmed Robotic Surgery, 93073 Neutraubling, Germany; 3 Regensburg Center of Health Sciences and Technology (RCHST), OTH Regensburg, 93053 Regensburg, Germany; 4 Regensburg Center of Biomedical Engineering (RCBE), OTH Regensburg and Regensburg University, 93053 Regensburg, Germany

**Keywords:** Generalized zero-shot learning, Sentence embeddings, Z-score normalization, Multi-label classification, Surgical instruments

## Abstract

**Purpose:**

Recognizing previously unseen classes with neural networks is a significant challenge due to their limited generalization capabilities. This issue is particularly critical in safety-critical domains such as medical applications, where accurate classification is essential for reliability and patient safety. Zero-shot learning methods address this challenge by utilizing additional semantic data, with their performance relying heavily on the quality of the generated embeddings.

**Methods:**

This work investigates the use of full descriptive sentences, generated by a Sentence-BERT model, as class representations, compared to simpler category-based word embeddings derived from a BERT model. Additionally, the impact of z-score normalization as a post-processing step on these embeddings is explored. The proposed approach is evaluated on a multi-label generalized zero-shot learning task, focusing on the recognition of surgical instruments in endoscopic images from minimally invasive cholecystectomies.

**Results:**

The results demonstrate that combining sentence embeddings and z-score normalization significantly improves model performance. For unseen classes, the AUROC improves from 43.9 % to 64.9 %, and the multi-label accuracy from 26.1 % to 79.5 %. Overall performance measured across both seen and unseen classes improves from 49.3 % to 64.9 % in AUROC and from 37.3 % to 65.1 % in multi-label accuracy, highlighting the effectiveness of our approach.

**Conclusion:**

These findings demonstrate that sentence embeddings and z-score normalization can substantially enhance the generalization performance of zero-shot learning models. However, as the study is based on a single dataset, future work should validate the method across diverse datasets and application domains to establish its robustness and broader applicability.

**Supplementary Information:**

The online version contains supplementary material available at 10.1007/s11548-025-03439-5.

## Introduction

Minimally invasive surgery (MIS) and robotic-assisted surgery (RAS) have revolutionized modern surgical practices due to their numerous advantages over traditional surgery methods [1–3]. These include reduced recovery times, less postoperative pain, and lower infection risks [1]. RAS further enhances surgical precision through features like improved ergonomics, tremor reduction, and 3D visualization, enabling more efficient workflows and advanced autonomous functionalities [2].

To fully leverage these advancements, integrating intelligent systems into surgical environments has become increasingly important. Deep learning (DL) has emerged as a powerful tool for computer vision applications, enabling the analysis and interpretation of complex visual data captured during medical procedures [4, 5]. DL-based systems, for instance, can automatically recognize and track surgical instruments, a critical capability of optimizing robotic workflows, enhancing surgical decision-making, and ensuring patient safety. However, deploying these systems in real-world scenarios faces significant challenges.

A key obstacle, especially in the medical domain, is the lack of large and diverse data sets for the training of robust DL systems. In surgical settings, this is even more complicated by significant visual variability, including overlapping instruments, smoke, blood, image blur, or reflections [6]. Furthermore, the frequent introduction of new or vendor-specific instruments adds to the complexity, often rendering traditional supervised learning approaches insufficient for generalization across unseen instruments.

Zero-shot learning (ZSL) directly addresses these limitations by enabling classification of instruments that were not encountered during training. ZSL reduces the dependency on exhaustive labeled datasets and facilitates adaptability across diverse surgical settings. This makes ZSL particularly valuable in high-stakes scenarios, where robust and scalable systems are essential. Generalized zero-shot learning (GZSL) extends this capability by enabling classification of both seen and unseen classes in inference [7]. These approaches are particularly valuable in endoscopic instrument recognition, where new instruments are regularly introduced and need to be reliably identified.

In this work, we propose a novel approach to enhance the generalization capabilities of ZSL in the context of endoscopic surgical instrument classification. Traditional methods often rely on semantic vector representations derived from individual words [8, 9], which provide limited contextual information about a class. To address this, we investigate the impact of using complete descriptive sentences to represent instrument categories. These sentence embeddings, generated by the Sentence-BERT (SBERT) model [10], are compared to word embeddings derived from class names using BERT. Additionally, we explore the role of post-processing techniques in improving embedding quality. Specifically, we evaluate the impact of z-score normalization on both the contextualized sentence embeddings generated by SBERT and the word embeddings derived from BERT.

## Related work

**Generalized Zero-Shot Learning:** GZSL methods can be categorized based on the availability of information about unseen classes during training and the techniques used to enrich models with semantic information. In transductive learning, visual features of unseen classes are available during training but remain unlabeled, while inductive learning excludes any visual representation of unseen classes from the training data [11]. Two primary approaches dominate GZSL: generative and embedding-based methods. Generative approaches create synthetic, visual features for unseen classes to frame the task as a supervised problem alongside the seen classes. Common implementations include conditional Wasserstein generative adversarial networks (cWGAN) [12], diffusion models [13], or hybrid models combining variational auto-encoders (VAEs) with GANs [14]. In contrast, embedding-based methods aim to map visual features and semantic embeddings in a shared space. Recognition of unseen classes relies on a projection function and similarity-based inference using prototypical representations  [7, 15].

**Semantic Embeddings:** Semantic information is critical for GZSL, with two main sources being the manually defined attributes [16] and word embeddings [17]. Attributes often describe visual characteristics such as shape or color, while word embeddings are typically generated using models like word2vec [8], GloVe [9], or BERT [18]. To overcome the limitations of word-level embeddings, SBERT [18] extends BERT by introducing Siamese and triplet network architectures. This enables the generation of sentence-level embeddings optimized for semantic similarity tasks, a significant improvement over standard BERT embeddings for sentence-based applications.

**Fig. 1:**
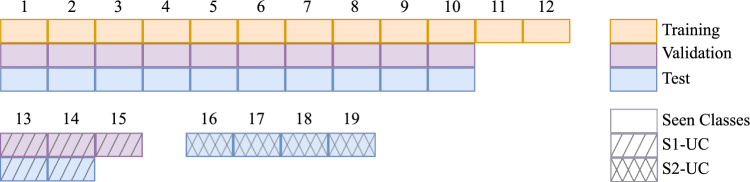
Used data split for the PhaKIR dataset: The training set comprises 12 seen classes derived from four videos, all sourced from the same hospital. In the validation phase, 10 of these seen classes are retained, and three novel unseen classes, referred to as stage one unseen classes (S1-UC), are introduced. The test set includes the 10 seen classes, two of the three S1-UC, and four new unseen classes, designated as stage two unseen classes (S2-UC). The two videos for validation and testing each come from a known and an unknown hospital

**Embedding Normalization:** Normalization techniques play a vital role in improving embedding quality and addressing specific challenges in high-dimensional spaces. For instance, z-score normalization has been identified as a key post-processing step in lexical tasks such as word similarity [19]. This technique centers and scales embedding values to a mean of zero and a standard deviation of one, enhancing their discriminative power. Another line of research has applied normalization on the feature level of neural networks to mitigate the hubness problem [20], where certain prototypes dominate as nearest neighbors, impairing recognition performance [21]. Despite these advancements, existing research on z-score normalization has primarily focused on word embeddings or feature normalization within networks. Its impact on the generalization capability of GZSL methods utilizing sentence embeddings remains largely unexplored. This work addresses this gap by evaluating the role of z-score normalization in conjunction with SBERT-generated sentence embeddings.

## Dataset

The dataset used in this study is derived from the Surgical Procedure Phase Recognition, Keypoint Estimation, and Instrument Instance Segmentation (PhaKIR) Challenge,^1^ which was held in 2024 as part of the Medical Image Computing and Computer Assisted Intervention (MICCAI) conference [22]. The publicly available training data includes eight videos from three hospitals, showcasing minimally invasive cholecystectomies and representing 19 different instrument classes. In our work, we focus exclusively on the published subset of the dataset, using only frames where instruments are visible. This amounts to 14,620 frames out of a total of 19,435. To align with the inductive GZSL framework, the dataset was split based on instrument classes and hospital origins as shown in Fig. 1.

Three instruments (*Argonbeamer*, *Hook-Clamp*, and *Bipolar-Clamp*) posed unique challenges due to the absence of ground truth annotations in the validation or test splits. While the Argonbeamer and Hook-Clamp are seen classes, and the Bipolar-Clamp is a stage one unseen class (S1-UC), we included them in the training and validation phase, respectively, to leverage their visual representations and semantic embeddings for improved generalization. However, they were excluded from the validation and testing phases accordingly.

This dataset presents two key challenges for GZSL models: unseen class recognition and domain adaptation. Our dataset setup ensures a rigorous evaluation of both aspects. The introduction of entirely novel classes and hospital environments tests the model’s ability to generalize across diverse scenarios.

## Methods

Our proposed method is presented in three parts below. First, the baseline approach, which serves as the foundation of our experiments. Next, we detail the integration of phrase-based embeddings using SBERT, and finally, we discuss the application of z-score normalization to enhance embedding quality.

### Baseline approach

**Fig. 2:**
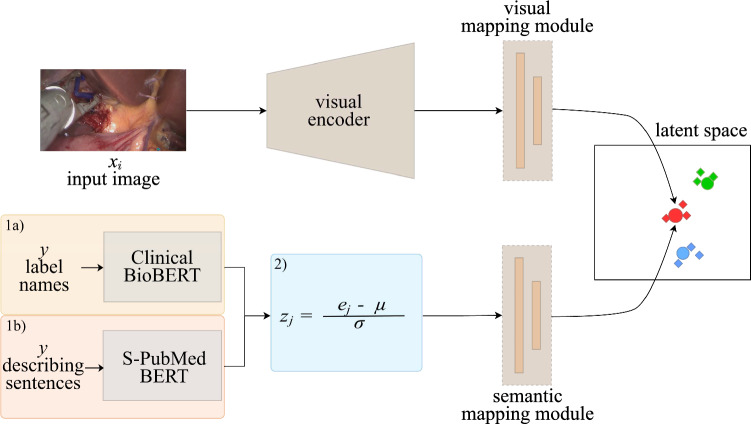
Our proposed GZSL Architecture. Steps 1a) and 1b) show either the word input embeddings created with Clinical BioBERT or the sentence input embeddings created with S-PubMedBERT. Step 2) shows the optional z-score normalization as a post-processing step on the input embeddings for the remaining pipeline, which is omitted in the Baseline approach

The baseline method in this work, illustrated in Fig.  2, builds on the framework introduced by Hayat et al in [15], which implements a multi-label GZSL approach for disease classification in chest radiographs. This method was selected due to its proven effectiveness in handling multi-label GZSL tasks, making it a suitable starting point for surgical instrument recognition.

The original framework employs DenseNet-121 [23] as the backbone for feature extraction, followed by a visual mapping module with three fully connected layers. In parallel, a semantic mapping module, also consisting of three fully connected layers, processes word embeddings generated by BioBERT [24]. Both visual and semantic features are mapped into a shared embedding space, enabling label predictions. The model optimizes three loss functions:

1. Ranking loss: Prioritizes relevant labels by ranking them higher than irrelevant ones.
2. Alignment loss: Ensures alignment between visual and semantic features in the shared latent space.
3. Semantic inter-class consistency loss: Preserves inter-class relationships from the original word embeddings to the latent space.

For our work, this framework was adapted to the context of surgical instrument recognition in minimally invasive procedures. Several modifications were made to tailor the approach to the dataset and task requirements. First, instead of BioBERT, we employed Clinical BioBERT [25], a model fine-tuned on clinical text from the MIMIC-III v1.4 database [26]. This adjustment leverages domain-specific semantics to improve model performance. Second, the number of feedforward layers in the visual and semantic mapping modules was reduced from three to two. This modification balances model complexity and generalization, addressing the smaller dataset and minimizing the risk of overfitting.

### Phrase-based embeddings with SBERT

Using descriptive phrases instead of simple label names for instrument embeddings is motivated by the need for deeper semantic information to enhance generalization to unseen classes. In the GZSL framework, visually similar representations are expected to cluster closely in the latent space, while semantically similar embeddings should align similarly. Instrument names alone often fail to capture this semantic richness. Many names are either ambiguous or non-descriptive of an instrument’s function or appearance. By incorporating detailed descriptions, we aim to reduce these ambiguities and improve the embedding alignment in both the original and latent spaces. To generate these descriptive sentences, we utilized GPT-4,^2^ prompting it with contextual information about minimally invasive cholecystectomy and the need to describe each instrument’s function and appearance. The exact procedure for creating the embeddings can be found in Online Resource 1. While GPT-4 performed well for most instruments, manual adjustments were required for some cases to ensure accuracy. The final descriptions were embedded using the fine-tuned S-PubMedBERT model,^3^ which is specially trained on clinical data, including PubMed abstracts and full-text articles from PubMedCentral.

**Fig. 3:**
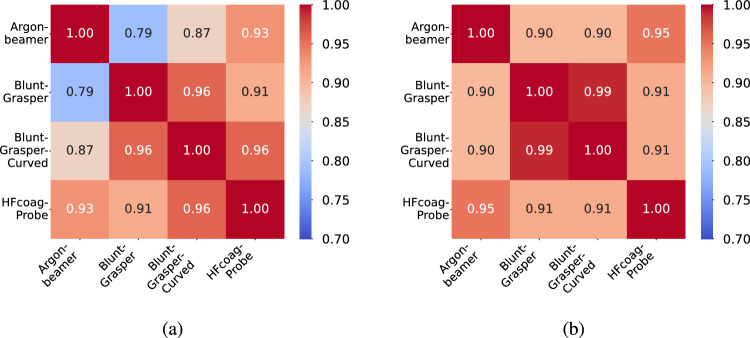
Cosine similarity scores for contextualized word embeddings created by Clinical BioBERT **a** as well as for contextualized sentence embeddings created by S-PubMedBERT **b** without z-score normalization

To assess the quality of the generated embeddings, we computed the cosine similarity between all embedding pairs. Figure 3 illustrates the relationships for specific classes: *Argonbeamer*, *Blunt-Grasper*, *Blunt-Grasper-Curved*, and *HFcoag-Probe*.

In Fig. 3a, using word embeddings, the *HFcoag-Probe* shows higher similarity to the *Blunt-Grasper-Curved* than to the *Argonbeamer*. This contradicts their functional similarity, as both *HFcoag-Probe* and *Argonbeamer* are designed to coagulate tissue. Conversely, Fig. 3b, using sentence embeddings, more accurately reflects these functional relationships, showing a higher similarity between *HFcoag-Probe* and *Argonbeamer* than between *HFcoag-Probe* and *Blunt-Grasper-Curved*. This adjustment is logical since the Blunt-Grasper and its curved variant are primarily used for grasping tissue. These observations indicate that sentence embeddings more effectively capture functional, and visual relationships, overcoming the limitations of simple word embeddings. This serves as the foundation for subsequently investigating their impact on generalization ability.

### Embedding normalization

A closer inspection of Fig.  3 reveals that while the relative relationships between embeddings are well preserved, the absolute cosine similarity values are confined to a narrow range despite the theoretical range of $[-1,1]$. This raises the question of whether normalization techniques, such as z-score normalization, can enhance embedding quality by expanding the value range. To explore this, we applied z-score normalization to the embeddings as a post-processing step. This transformation ensures a mean of zero and a standard deviation of one, as defined in Equation 1.

1 $$\begin{aligned} z_j = \frac{e_j - \mu }{\sigma } \end{aligned}$$

where

- $z_j$ represents the normalized embedding,
- $e_j$ is the original embedding,
- $\mu$ represents the mean, and
- $\sigma$ is the standard deviation of the embeddings.

**Fig. 4:**
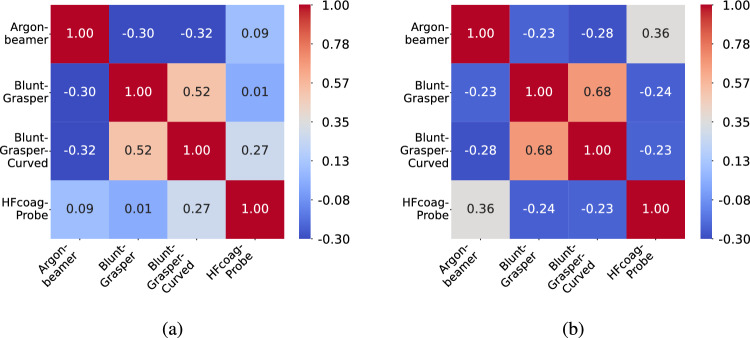
Cosine similarity scores for contextualized word embeddings created by Clinical BioBERT **a** as well as for contextualized sentence embeddings created by S-PubMedBERT **b** with z-score normalization

This process was applied to both word and sentence embeddings to evaluate its impact on cosine similarity relationships. Figure 4 compares the cosine similarities of normalized word embeddings (Fig. 4a) with their normalized sentence embedding counterparts (Fig. 4b). After normalization, the cosine similarity values span a wider range while maintaining the relative relationships observed in Fig.  3.

The expansion of the value range has practical implications for GZSL tasks. By spreading the embedding distances more evenly across the similarity spectrum, normalization may help the model better differentiate between classes, particularly in high-dimensional spaces prone to the hubness problem, where certain embeddings dominate as nearest neighbors [21].

In the next section, we analyze how this normalization affects the generalization capability, focusing on its ability to classify unseen classes accurately.

## Experiments and results

All experiments in this study were based on an adaptation of the codebase from [15]. The vision backbone employed was DenseNet-121, initialized with random weights, and all input images were resized from $1920 \times 1080$ to $224 \times 224$. Training was optimized using the Adam optimizer [27], running for 50 epochs with a batch size of 16. A dynamic learning rate adjustment was implemented via the OneCycleLR scheduler [28] with a maximum learning rate of 1e-4. Most hyperparameters were adopted from [15], except for the margin value in the ranking loss, which was adjusted to $\delta = 0.3$ based on preliminary experiments.

The experiments were conducted on NVIDIA GeForce RTX 3090 and A100 80GB PCIe GPUs. To evaluate the robustness of our model’s performance, we performed 25 independent runs per experiment, each with a different random seed. This resulted in a unique resampling of the training dataset with replacement, while maintaining the same total number of samples. Each trained model was then evaluated on the same test dataset. This approach enables us to quantify the variability in model performance and assess its generalization stability more effectively.

Model performance was evaluated using the macro-F1 score, area under the receiver operating characteristic curve (AUROC)—both common multi-label performance metrics [29]—and a dynamic multi-label accuracy metric.^4^ The model selection criterion was the highest macro-F1 score achieved on the validation data, which includes both seen and unseen (S1-UC) classes. By incorporating unseen classes into the validation set, the model’s generalization ability was directly assessed during training. To ensure robust evaluation on unseen classes, new classes were reserved exclusively for the test split (S2-UC). For threshold-independent comparisons, we report both mean AUROC and multi-label accuracy values.

Unlike standard multi-label accuracy, which relies on exact matches (subset accuracy), we apply a dynamic top-*k* strategy, where *k* is equal to the number of ground truth labels for each instance. The model’s top-*k* predicted classes (based on the highest prediction scores) are then compared with the ground truth set. The accuracy is computed as the proportion of correctly predicted labels. In Equation 2, the multi-label accuracy is calculated as follows:

2 $$\begin{aligned} \text {Multi-Label Accuracy} = \frac{1}{N} \sum _{i=1}^{N} \frac{|P_i \cap T_i|}{|T_i|}, \end{aligned}$$

where $|T_i|$ indicates the total number of relevant (ground truth) classes for test instance $i$. $T_i$ denotes the set of relevant (ground truth) classes for test instance $i$, while $N$ represents the number of test instances with at least one relevant class ($|T_i| > 0$). $P_i$ is the set of the top-$|T_i|$ predicted classes with the highest scores for that instance. Finally, the term $|P_i \cap T_i|$ refers to the number of correctly predicted classes, for instance $i$, which is the intersection of predicted and relevant classes.

For example, if a test image has ground truth labels {A, B, C}, (i.e., $$ |T_i| = 3$$), and the model’s top-3 predictions are {A, D, B}, the intersection is {A, B}, resulting in a multi-label accuracy of $$2/3$$ for that instance. The overall multi-label accuracy is then obtained by averaging this accuracy over all instances, which provides a flexible and context-sensitive evaluation for multi-label classification tasks.

For both AUROC and multi-label accuracy, results were reported separately for seen, stage one, and stage two unseen classes. To provide a balanced assessment of the model’s overall performance, we also compute the harmonic mean across all three groups. This approach ensures that the model’s performance on unseen classes, which is critical in GZSL tasks, is given equal consideration alongside seen classes. During evaluation, predictions are made over the complete label set, but metric calculations for each group are performed by filtering the predicted and ground truth labels to include only the relevant class subset (e.g., only S2-UC labels when evaluating zero-shot performance). While we report metrics for all subsets and their harmonic mean, we place particular emphasis on the results for stage two unseen classes (S2-UC), as these represent the truly novel categories that were not seen during training or validation. All results are presented as the mean of 25 repeated runs.

**Table 1:**
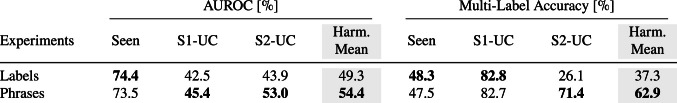
Comparison of AUROC and multi-label accuracy values for word and sentence embeddings for the baseline method

Bold values indicate better results

### Word vs sentence embeddings

In our first experiment, we evaluated the performance of word embeddings (*Labels*) and sentence embeddings (*Phrases*) within the baseline model setup. The results, as summarized in Table 1, highlight significant improvements when using sentence embeddings, especially for the S2-UC.

For the AUROC metric, sentence embeddings improved performance by 9.1 percentage points for the unseen test classes and by 5.1 percentage points for the harmonic mean. For the multi-label accuracy, we observed a substantial improvement of 45.3 percentage points for unseen test classes and an increase of 25.6 percentage points for the harmonic mean, while the results for the seen classes remained largely unchanged for both metrics. These findings establish that sentence embeddings provide a significant advantage over word embeddings in the context of multi-label classification tasks. The use of descriptive phrases instead of single-label names offers clear benefits, as reflected in the improved performance for novel classes.

### Raw vs Z-score normalization on embeddings

In this experiment, we evaluated the impact of z-score normalization on both word (*Labels*) and sentence (*Phrases*) embeddings. Table 2 summarizes the results, comparing the unnormalized (*Raw*) embeddings and their z-score normalized (*Z-Score*) counterparts. The experimental pipeline, as illustrated in Fig. 2, follows path 1a) and 1b) for raw embeddings and path 1a) + 2) and 1b) + 2) for normalized embeddings. Applying z-score normalization improved model performance particularly for the AUROC metric, across both embedding types.

**Fig. 5:**
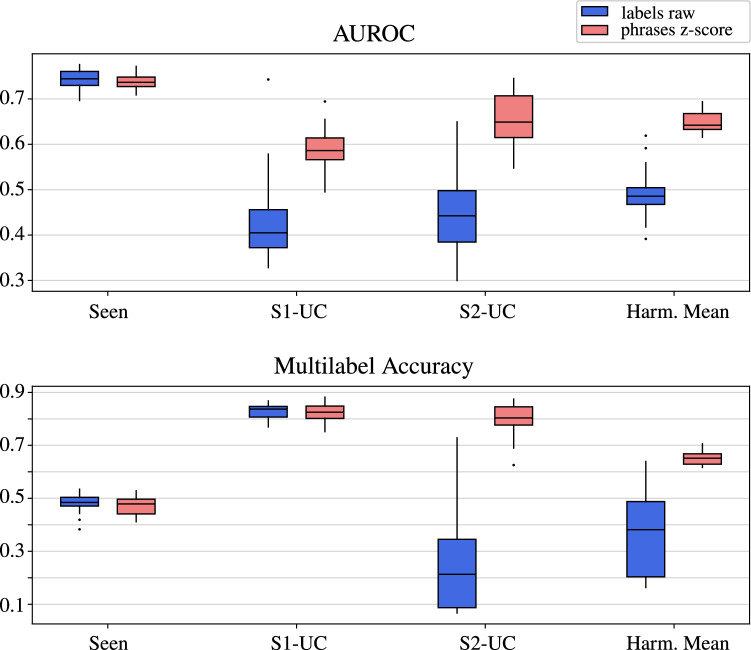
Box plot comparison for replicate experiments with the label embedding (blue) vs. the z-score normalized phrase embedding (red). Results are compared for seen classes, S1-UCs, S2-UCs and the harmonic mean

**Results for Word Embeddings.** AUROC values increased by 4.8 and 5.5 percentage points for the unseen test classes and the harmonic mean, respectively. Multi-label accuracy values deteriorated by 4.7 and 2.7 percentage points for the unseen test classes and the harmonic mean, respectively.

**Results for Sentence Embeddings.** There was a 11.9 percentage point increase in AUROC for the unseen test classes and a 10.5 percentage point rise for the harmonic mean. Additionally, multi-label accuracy values improved by 8.1 and 2.2 percentage points for the same categories.

**Fig. 6:**
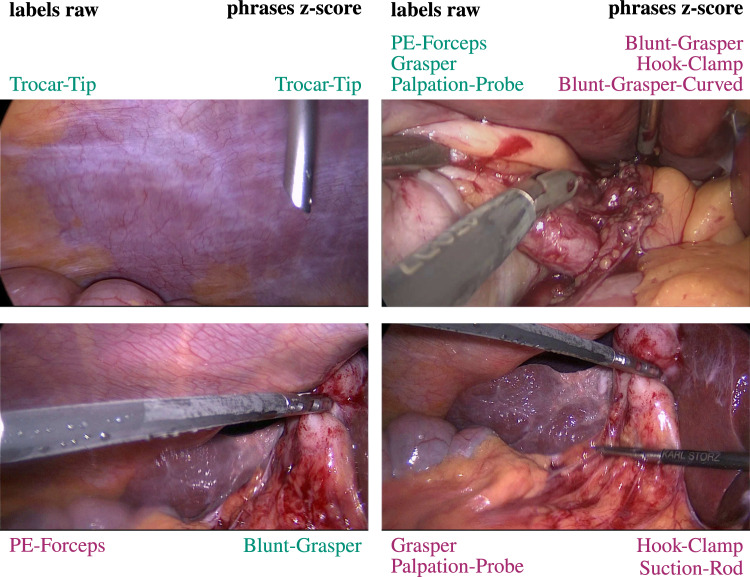
Comparison of model predictions using two different embedding types: raw label embeddings and z-score normalized phrase embeddings. Correct predictions are highlighted in green, while incorrect ones are shown in purple. The results reveal a balanced distribution across the four examples: one instance shows correct predictions from both methods, another shows failures from both, while the remaining two display divergent performances between the methods

These results demonstrate that z-score normalization has a substantial positive impact on both embedding types, with particularly pronounced improvements for unseen test classes, especially in terms of the AUROC metric. Meanwhile, the results for seen classes remain largely unchanged, indicating that the normalization primarily benefits generalization to unseen data.

To further assess the model’s robustness concerning these performance improvements, Fig. 5 presents box plots comparing the raw label embeddings with the z-score normalized phrase embeddings. The boundaries of the boxes represent the $\text {25}^{\text {th}}$ and $\text {75}^{\text {th}}$ percentiles of the replicate experiments, while the line within the box indicates the median. As the previous results showed, there is little difference for the seen classes, whereas the unseen classes exhibit a substantial improvement. In addition, it can be observed that the variance of the results is rather low for the seen classes, but significantly higher for the unseen classes. However, the use of z-score normalized phrase embeddings was able to mitigate this significantly in some cases.

**Table 2:**
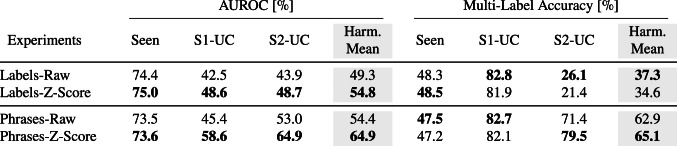
Comparison of AUROC and multi-label accuracy values for word and sentence embeddings, highlighting the impact of the z-score normalization

Bold values indicate better results

Figure 6 illustrates the model predictions for four sample images using two different embedding types: raw label embeddings and z-score phrase embeddings. The results demonstrate a balanced distribution across the examples: in one case, both methods yield correct predictions; in another, both fail; and in the remaining two cases, performance varies between the methods.

### Ablation study

To further investigate the generalization ability of our approach, we conducted an ablation study examining the impact of using a shared versus separate optimization strategy for the loss functions.

In the baseline method, all loss functions—ranking, alignment, and inter-class consistency—are combined into a single-gradient descent update. However, since these loss functions have distinct objectives, we hypothesized that separating their optimization might yield better results.

In the multiple optimizer approach, the model’s parameters for the visual encoder and the visual mapping module are updated using a single-gradient descent step, driven by the combined ranking and alignment losses. Meanwhile, the semantic mapping module’s parameters are refined in a separate gradient descent step, guided by the inter-class consistency loss. Table 3 compares the performance of the baseline method (shared optimization) with the multiple optimizer approach for both word and sentence embeddings, with z-score normalization applied in all cases.

Contrary to our initial assumption, the results reveal minimal differences between the optimization types, indicating that our hypothesis is not supported by the observed outcomes. These findings suggest that regardless of the embedding type, the choice of optimization strategy has negligible impact on the model’s generalizability.

**Table 3:**
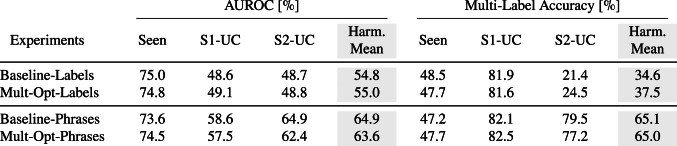
Comparison of AUROC and multi-label accuracy values between the baseline approach and the multiple optimizer setting for both word and sentence embeddings with z-score normalization

## Discussion

The results presented in Chapter Word vs sentence embeddings support our hypothesis that using complete descriptive sentences for class representation enhances generalization compared to simple category labels. Both AUROC and multi-label accuracy consistently improve with sentence embeddings, particularly for unseen classes. This significant improvement underscores the strength of sentence embeddings in capturing richer semantic information, which better aligns with the visual and functional similarities of previously unseen classes. For seen classes, the results were less conclusive, suggesting that the benefits of sentence embeddings become more pronounced when the model is exposed to unfamiliar data.

The findings in Chapter Raw vs Z-score normalization on embeddings highlight the critical role of z-score normalization in improving overall model performance. Normalization yields notable gains in AUROC for both word and sentence embeddings, as well as for multi-label accuracy for the sentence embeddings. These improvements are most significant for unseen classes, indicating that normalization helps the model to better generalize by mitigating issues such as hubness and ensuring a more balanced representation in high-dimensional spaces, which aligns with previous research [21]. Furthermore, this normalization technique enhances the model’s robustness, reducing its dependence on specific training data distributions.

In the ablation study (Chapter Ablation study), the assumption that a decoupled loss calculation would enhance generalization was not confirmed.

While these findings provide valuable insights, the study has certain limitations. All experiments were conducted on a single dataset, which may limit the generalizability of the conclusions to other domains or datasets with different characteristics. Furthermore, hyperparameter choices and architectural settings—such as the use of two feedforward layers—were based on practical considerations rather than comprehensive optimization. Future studies could address these limitations by exploring alternative datasets, domains, or architectures. Moreover, integrating more advanced semantic models or unsupervised techniques for embedding generation may further enhance performance and robustness.

## Conclusion

This work investigated the use of descriptive sentences as embeddings, compared to simple labels, to improve the generalization capability in surgical instrument classification on laparoscopic endoscopic images. We also evaluated the impact of z-score normalization as a post-processing technique. The results showed that sentence embeddings consistently outperform word embeddings, particularly on unseen classes, validating our initial hypothesis. Furthermore, applying z-score normalization significantly enhanced the model’s ability to generalize, highlighting its value as a key component in GZSL pipelines.

These findings highlight the potential of enriched semantic representations and normalization techniques for improving performance in multi-label classification tasks. Future research should expand on this approach by exploring its applicability to other datasets, domains, or tasks beyond surgical instrument classification. Investigating alternative architectures, optimization strategies, or more advanced embedding generation methods could further improve robustness and scalability in diverse real-world scenarios.

## Supplementary Information

Below is the link to the electronic supplementary material.

Supplementary file 1 (pdf 194 KB)

## Footnotes

1 https://phakir.re-mic.de/wp-content/uploads/2024/07/PhaKIR_Data_Description_and_Labeling_Instructions_v2.pdf

2 https://openai.com/index/gpt-4/

3 https://huggingface.co/pritamdeka/S-PubMedBert-MS-MARCO

4 https://pytorch.org/torcheval/main/generated/torcheval.metrics.MultilabelAccuracy.html
