# Supplementary material for: Enhancing generalization in zero-shot multi-label endoscopic instrument classification
Source: Int J Comput Assist Radiol Surg. 2025 Jun 11;20(8):1577–87. doi: 10.1007/s11548-025-03439-5 (PMC12350423; doi:10.1007/s11548-025-03439-5)
Supplement: Supplementary file 1 — (pdf 194 KB) [file 11548_2025_3439_MOESM1_ESM.pdf]

International Journal of Computer Assisted Radiology and Surgery

Enhancing Generalization in Zero-Shot Multi-Label Endoscopic Instrument Classification

Raphaela Maerkl[1*], Tobias Rueckert[1,2], David Rauber[1], Max Gutbrod[1,3], Danilo Weber Nunes[1,3], Christoph Palm[1,3,4]

[1]Regensburg Medical Image Computing (ReMIC), OTH Regensburg, Regensburg, 93053, Germany.
[2]AKTORmed Robotic Surgery, Neutraubling, 93073, Germany.
[3]Regensburg Center of Health Sciences and Technology (RCHST), OTH Regensburg, Regensburg, 93053, Germany.
[4]Regensburg Center of Biomedical Engineering (RCBE), OTH Regensburg and Regensburg University, Regensburg, 93053, Germany.

[*]Corresponding author(s). E-mail(s): raphaela.maerkl@st.oth-regensburg.de

Online Resource 1: Creation of Sentence Embeddings

In this chapter, the process of creating sentence embeddings will be explained in greater detail. We began by providing GPT-4 with a description of the problem context, which is as follows:

> *I would like to train a generalized zero-shot learning network. For this purpose, I have a vision backbone that extracts the visual features of the input image. These extracted features are then passed through three fully connected layers (visual mapping). Additionally, I embed a fixed text representation using a fine-tuned BERT model. This embedding (the CLS token) is also passed through three fully connected layers (text mapping). The dimensions of the visual and text mappings are identical in each case.*
>
> *From this point, I compute various losses to align the visual and textual features as closely as possible in the latent space. During training, I use fewer classes than during testing. This means that during inference, I encounter images of completely unseen classes, which I aim to classify solely based on text embeddings (where all classes, both seen and unseen, are included). My goal is to recognize both seen and unseen classes during inference.*
>
> *The classification task is multi-label, meaning that multiple classes can be present in a single image. The application focuses on recognizing surgical instruments in minimally invasive procedures, such as gallbladder removal.*
>
> *In the following, I will provide a list of different instrument names that represent my classes. I would like you to generate a brief descriptive paragraph for each instrument, detailing its function and appearance as accurately as possible. If you are unfamiliar with any of the instruments or their appearance, please let me know.*
>
> *My goal is to use these descriptions to generate text embeddings while preserving contextual information.*
>
> *The classes are as follows:*

['Argonbeamer', 'Bipolar-Clamp', 'Blunt-Grasper', 'Blunt-Grasper-Curved', 'Blunt-Grasper-Spec.', 'Clip-Applicator', 'Dissection-Hook', 'Drainage', 'Grasper', 'HFcoag-Probe', 'Hook-Clamp', 'Needle-Probe', 'Overholt', 'Palpation-Probe', 'PE-Forceps', 'Scissors', 'Sponge-Clamp', 'Suction-Rod', 'Trocar-Tip']

As a response, we received several descriptive sentences for each class, which varied significantly in quality. For this reason, there were several exchanges to refine the descriptions and ensure their accuracy. We explicitly provided corrections for specific instruments, as shown below:

The 'Sponge Clamp' does not feature long jaws; they are typically short. Additionally, the hook of the 'Dissection-Hook' is relatively small.

Overall, the descriptions were satisfactory; however, we sought more tailored representations for the instrument classes. Therefore, we decided to combine our own knowledge of the instruments' functions and appearances with that of GPT-4, manually refining the sentences using the generated content.

The final descriptions are as follows:

Seen Classes

The **Argonbeamer** is used for haemostasis and tissue revitalization. Without contact, energy is transferred to the tissue by ionized argon gas (argon plasma). This allows tissue to be coagulated and bleeding to be controlled efficiently. The instrument has a long, rigid, cylindrical shape that ends in a black sheath, with the tip being white and rounded. The white tip marks the exit end for the argon gas and electric current, which produces a visible discharge when activated to treat the tissue precisely.

The **Clip-Applicator** is used to attach metal or plastic clips to vessels or structures to seal or close them. The instrument is usually metallic or made of dark plastic. It has a rather wide shaft and two movable pliers at the tip, in which the clip is clamped. There are notches at the foremost points of the tip to hold the clip. In some cases the tips are very narrow and small.

The **Drainage** tube is used at the end of an operation to remove gas that was introduced to inflate the abdomen for better visibility during surgery. It is flexible and features multiple small holes through which the gas is sucked out, ensuring the abdominal cavity is properly deflated post-procedure. The drainage is usually white or transparent and in some cases has a blue stripe along the tube.

The **Grasper** is a versatile tool used to hold, manipulate, or extract tissues or foreign objects during surgery. It has a long, grey shaft with a metallic tip. The gripping jaws are pointed towards each other and have a serration at the front tip to hold fabric very firmly. The jaws are smooth on the inside and have an angular form. It has a smooth, closed surface. A special feature here is that only the upper jaw can move and the lower jaw is firmly attached to the shaft.

The **HFcoag-Probe** is used to coagulate tissue in a targeted manner using high-frequency current, which can effectively stop bleeding and revitalize tissue. The instrument has a long, cylindrical gray shaft with a slightly rounded tip, which is the active end. The tip is metallic and has the shape of a hemisphere.

The **Hook-Clamp** is used to grip or manipulate tissues or vessels securely during surgery. It is a clamping instrument with two curved, hook-like jaws, both tips mirroring each other. The hook-shaped jaws, combined with its long handle, offer reliable tissue manipulation, particularly for tasks that require a firm and secure hold. The metal instrument has fine serrations on the inside of the forceps to hold the tissue well. The clamp jaws are slightly more bulbous.

The **Needle-Probe** is a fine, metallic, needle-shaped instrument primarily used to puncture the abdominal wall, making it easier to insert the trocar tip for access. The probe features a sharp, slender

tip for creating a clean entry point, while its long handle provides precise control, ensuring minimal tissue trauma during insertion.

The **Palpation-Probe** is used to gently palpate tissue, or to hold organs and other tissue structures away for a better view. It consists of a long, round, metallic rod. The rod is alternately matt and shiny metallic to better assess the depth of penetration. There is a blunt rounding at the foremost point of the rod to avoid damaging the tissue.

The **PE-Forceps** is used to expose organs, grasp tissue, manipulate or hold it precisely. The instrument has a long, gray shaft with a metallic tip. To make it easier to cut through the tissue, an electric current is usually applied. The jaws have an oval shape at the tip with a medium-sized hole at the top. The jaws also have a rather bulbous shape.

Surgical **scissors** are used to cut the bile ducts and possibly other blood vessels. The instrument has two metal and curved cutting jaws that are smooth and sharp on the inside to ensure a clean cut. It is finely serrated along one edge. Electricity can also be used to cut through the tissue more easily. It also has a long, round and gray shaft.

The **Suction-Rod** is a dual-purpose instrument used both to suction fluids and introduce water for rinsing during surgery. The instrument is essential for maintaining a clear view during surgery by removing blood or debris and assisting with rinsing. It has a long, cylindrical and metallic body with a suction tip for removing fluids, and a channel for introducing water to flush the surgical site. It has few very small holes along the tip to allow the liquids to be sucked in and out. The tip has a blunt and open ending.

The **Trocar-Tip** is the pointed end of a trocar, which is used to create an access port during surgeries. The sharp tip pierces the skin and underlying tissues, allowing for the insertion of instruments into the body cavity. It is designed to create a clean, controlled entry point, facilitating the introduction of surgical tools. It also describes the trocar tube through which the instruments are inserted into the abdominal cavity. It has an opening on both sides. The instrument is shiny metallic and has a smooth surface. In rare cases, it may also have a roughly grooved surface.

Stage One Unseen Classes

The **Bipolar-Clamp** is used to grip tissue in a targeted manner and simultaneously stop bleeding through bipolar coagulation. The instrument has a long, black, cylindrical shaft that merges into a white sheath for insulation. The tip consists of two movable and curved gripping jaws which become narrower towards the tip. It has fine serrations on the inner surfaces to hold the tissue firmly and coagulate it precisely.

The **Blunt-Grasper-Spec.** is used to grip and manipulate tissue and is specially adapted for precision tasks. The instrument has a long, narrow shaft. The gripping jaws are metallic, narrow and characterized by a hole. They are bulbous rather than flat. The inside of the jaws are serrated at the front to ensure a secure grip.

The **Dissection-Hook** is used to precisely separate or mobilize tissue. For example, organs can be exposed or separated from other tissue structures. The instrument has a long shaft, which is coated in black at the front. The metal tip is bent like a hook, approximately 90 degrees. It is very narrow and sharp at the tip and can therefore separate the tissue either by its sharp hook-like tip or by applying an electric current.

Stage Two Unseen Classes

The **Blunt-Grasper** is used to grasp and manipulate tissue without damaging it. It has a grey, cylindrical shaft with two moving metal jaws at the tip. The flat jaws for gripping are very long, straight, and have

a rounded tip. There are very fine serrations on the inside of the jaws to hold the tissue firmly. Along the jaws there are usually one or two large holes.

The **Blunt-Grasper-Curved** is used to gently grip and manipulate tissue, especially in hard-to-reach areas. The instrument has a long, grey and cylindrical shaft. The long tip has wide, flat jaws, which are bent at a certain angle in the front part. The metal jaws are also characterized by two large holes. They are finely serrated on the inside to hold the tissue firmly without damaging it. The tip is therefore also rounded at the foremost point.

The **Overholt** is used to grip, clamp or manipulate tissue or vessels. The instrument has a long, gray shaft with a metallic clamp as a tip, which can be spread open. The clamp jaws are bent by about 90 degrees. There is a fine ribbing on the inside to guarantee a good grip. The clamp jaws are slightly flatter.

The **Sponge-Clamp** is used for holding or positioning sponges during surgery, especially in cases where bleeding needs to be controlled. It has short, rounded, or serrated jaws that securely grip sponges or gauze without slipping. The compact jaws allow for easy manipulation of absorbent materials to soak up fluids during the procedure. Both the shaft and the tip are metallic. The round shaft is slightly wider than the tip.
